# Fast-Response Colorimetric UVC Sensor Made of a Ga_2_O_3_ Photocatalyst with a Hole Scavenger

**DOI:** 10.3390/s21020387

**Published:** 2021-01-08

**Authors:** Heejoong Ryou, Sunjae Kim, Myunghun Shin, Junsang Cho, Wan Sik Hwang

**Affiliations:** 1 Department of Materials Engineering, Korea Aerospace University, Goyang 10540, Korea; ryouheejoong@gmail.com (H.R.); kau.sjkim@gmail.com (S.K.); 2 Smart Drone Convergence, Korea Aerospace University, Goyang 10540, Korea; 3 School of Electronics and Information Engineering, Korea Aerospace University, Goyang 10540, Korea; mhshin@kau.ac.kr; 4 Department of Chemistry, Duksung Women’s University, Seoul 01369, Korea

**Keywords:** Ga_2_O_3_, colorimetric sensor, hole-scavenger, photocatalyst, ultraviolet-C

## Abstract

A fast-response colorimetric ultraviolet-C (UVC) sensor was demonstrated using a gallium oxide (Ga_2_O_3_) photocatalyst with small amounts of triethanolamine (TEOA) in methylene blue (MB) solutions and a conventional RGB photodetector. The color of the MB solution changed upon UVC exposure, which was observed using an in situ RGB photodetector. Thereby, the UVC exposure was numerically quantified as an MB reduction rate with the *R* value of the photodetector, which was linearly correlated with the measured spectral absorbance using a UV-Vis spectrophotometer. Small amount of TEOA in the MB solution served as a hole scavenger, which resulted in fast MB color changes due to the enhanced charge separation. However, excessive TEOA over 5 wt.% started to block the catalytical active site on the surface of Ga_2_O_3_, prohibiting the chemical reaction between the MB molecules and catalytic sites. The proposed colorimetric UVC sensor could monitor the detrimental UVC radiation with high responsivity at a low cost.

## 1. Introduction

Germicidal lamps emitting ultraviolet-C (UVC) radiation at a central wavelength of 254 nm are widely used in various places ranging from residential to medical buildings for air and water disinfection, as well as in the food industry to prolong the shelf life of foods and beverages [1,2]. Besides the use of germicidal UVC light to inactivate microorganisms, including bacteria, fungi, yeast, and viruses, UVC radiation can also be utilized for satellite and space communications, environmental monitoring, and rewritable data storage [3,4,5].

As the UVC light is widely used, it is important to monitor UVC irradiation increases for safety and exposure management because exposure to UVC light negatively affects DNA in the skin and eyes, leading to genetic damage and mutations [6,7,8]. It should also be noted that the UVC radiation effect on humans is cumulative; thereby it is also important to monitor the cumulative radiation dose [9]. All these necessitate the need for a UVC sensor that is capable of the quantification of accumulated UVC doses with fast responsivity. In general, UVC sensors can be classified into two categories based on the UVC detection mechanism: photoelectric sensors and photochromic sensors. Photoelectric sensors generate an electrical photocurrent, while the photochromic sensors change color in response to UVC exposure. Photoelectric sensors are advantageous in the real-time detection of UVC radiation in the presence of UVC light. On the other hand, photochromic sensors are good for monitoring an accumulated dose of UVC radiation.

Recently, colorimetric UVC sensors made of gallium oxide (Ga_2_O_3_) with an energy bandgap of about 4.9 eV have drawn increasing attention due to their low cost, simplicity, and ability to easily quantify an accumulated dose [9,10,11,12,13]. Although TiO_2_ (3.2 eV) and ZnO (3.4 eV) could be considered for photoelectrochemical reactions under UVC radiation, Ga_2_O_3_ (4.9 eV) showed a much better UVC sensitivity compared to TiO_2_ and ZnO [13]. These colorimetric UVC sensors are preferred for medical- and health-related products because the accumulated dose of UVC radiation can be visualized via a color change and thus is easily perceptible without expensive external equipment. However, the reported colorimetric sensor showed low responsivity and difficulty in quantitative recognition [10,11,12,13]. Thus, it is important to enhance the color-switching speed of the colorimetric UVC sensor and to express the color values numerically upon UVC exposure.

In this work, we developed a fast-response and low-cost MB-based colorimetric sensor made of a Ga_2_O_3_ photocatalyst by adding a small amount of hole scavengers and an RGB photodetector. The detecting capability of the UVC sensor was verified by comparing the measured spectral absorbance with a conventional ultraviolet-visible light (UV-Vis) spectrophotometer. Hole scavengers in the MB solution enhanced the spatial separation of photogenerated electron–hole pairs (EHPs). The developed UVC sensor can be used to monitor the UVC radiation dose in real time.

## 2. Experimental

### 2.1. Reduction of Methylene Blue (MB) with Ga_2_O_3_ Photocatalysts with 254 nm Radiation

Monoclinic *β*-phase Ga_2_O_3_ (99.995%) photocatalysts, purchased from CRM Material (Nanchang, Jiangxi, China), were used for a reduction of MB to leuco-MB (LMB). This photocatalytic reduction resulted in color changing from blue (MB) to colorless (LMB). Three milligrams of MB was dissolved in 400 mL of deionized (DI) water and a 100 mL MB solution (7.5 mg/L in DI water) was prepared. For each sample, 9 mg of Ga_2_O_3_ nanostructures were homogeneously dispersed in the 4 mL MB solution (2.25 mg/mL in DI water), which was then exposed under a 6 W UVC lamp (UVG-11, Analytik Jena, Jena, Germany) with a wavelength centered at 254 nm (1.38 mW/cm^2^) for different times. The degree of the color change in the MB solution could be used as an indicator of an accumulated dose of UVC radiation.

The MB solution naturally absorbs orange light well; therefore, the MB solution is observed in blue, the complementary color of orange. When exposed to UVC radiation, the MB solution is chemically reduced and absorbs the red light less such that the MB solution becomes transparent (colorless) over time because its color becomes indistinguishable [14]. The color change in the MB solution due to UVC exposure can be perceived directly and visually. The chemical reduction of the MB solution can be initiated by catalysts such as the Ga_2_O_3_ photocatalyst [15]. The Ga_2_O_3_ nanoparticle was in the form of nanorods whose average length was about 3 μm and whose surface area was 32.2 m^2^/g using the Brunauer–Emmett–Teller (BET) method [16]. The degree of the color change in the MB solution can be used as an indicator of UVC exposure, and in this work, we used the MB reduction rate (MBR) as a numerical indicator of the UVC exposure.

### 2.2. Triethanolamine (TEOA) as a Hole Scavenger

MB reduction is initiated by the photogenerated electron–hole pairs (EHPs) when the Ga_2_O_3_ is illuminated. The EHPs in the photocatalyst often recombine in the bulk and/or surface of the photocatalyst before the photocatalytic reaction. This charge recombination process degrades the photocatalytic reduction efficiency of the MB solution. To suppress the charge recombination process, TEOA (0–15 at.%) was added to the MB solution as a hole scavenger. The hole scavenger in the reaction system prolongs the electron lifetime by removing the holes used for the recombination in advance, and thereby provides sufficient time to initiate the photocatalytic process; as such, the reaction rate of the MB reduction can be enhanced [17].

### 2.3. Comparison of the Colorimetric UVC Detection Using UV-Vis Spectrophotometers and the R Value from the RGB Photodetector

**Figure 1:**
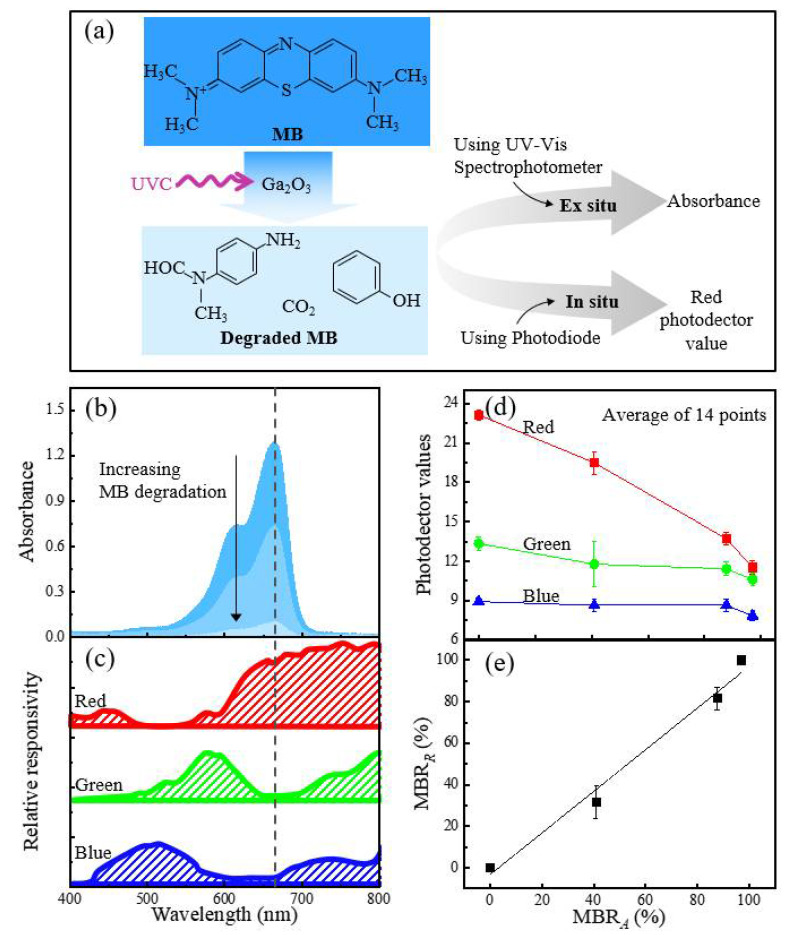
(**a**) Two different experimental setups for the ultraviolet-C (UVC) detection using methylene blue (MB) solution as a model system: MB reduction rates were quantified using either the ex situ absorption values from the UV-Vis spectrophotometer (MBR*_A_*) or the in situ *R* value from the RGB photodetector (MBR*_R_*). (**b**) Absorption spectra of the MB reduction with Ga_2_O_3_ photocatalysts at different UVC irradiation times. (**c**) The relative responsivity of the red (*R*), green (*G*), and blue (*B*) values in the range of 400 to 800 nm from the RGB photodetector datasheet [18]. (**d**) Extracted *R*, *G*, and *B* values from the RGB photodetector as a function of the MBR*_A_* values while the sample was under UVC illumination; the *R*, *G*, and *B* values were proportional to the given area of the *R*, *G*, and *B* relative responsivity, respectively, as seen in Figure 1c. (**e**) The correlation diagram between MBR*_A_* and MBR*_R_*. The average value along with the standard deviation for each point of the MBR*_R_* measurements was acquired using 14 different points.

To quantify the color changes, two approaches were conducted in this work and their reduction rate values were correlated with each other, as shown in Figure 1a. First, the color changes at the different UVC exposure times were quantified via the absorbance of the MB solution using a UV-Vis spectrophotometer (UV-3600 Plus, Shimadzu, Kyoto, Japan) at wavelengths in the range of 400–800 nm in Figure 1a,b. The absorption spectrum of the MB solution is characterized by two main absorption bands centered at 610 and 664 nm, which can be further assigned to the absorptions of the dimeric and monomeric forms of MB, respectively. The absorbance value at 664 nm was most sensitive to MB reduction; therefore, the value at 664 nm was monitored as a function of UVC exposure time.

The MB reduction rate (MBR*_A_*%) can be expressed with the absorbance value of the MB solution at 664 nm as (1):

(1) $${\mathrm{MBR}}_{A}=\frac{A_{0}-A_{t}}{A_{0}-A_{h}}\;\left(\%\right),$$

where *A*_0_ and *A_t_* are the initial absorbance and the absorbance at time *t* (min), respectively. *A_h_* is the absorbance of the DI water, which is almost equal to zero.

This approach using UV-Vis spectrophotometers can be called an ex situ colorimetric measurement. Although it can measure spectral change accurately, real-time monitoring is difficult because the measurement is made separately after the UV exposure experiment is completed.

The color change of MB solutions can be quantified in situ on an RGB color space by using an RGB photodetector (GY-31 TCS3200, Zhongshan Baijia Dagu Electronic Technology, Zhongshan, China). Figure 1c shows the relative spectral responsivity (red, green, and blue) of the photodetector that emulates the color-matching functions [14]. The measured outputs of the photodetectors represent *R*, *G*, and *B* values, respectively, which are overlapping integrals of the spectrum of a test light with each spectral responsivity of the photodetector in the spectral range of 400–800 nm. It reveals that the absorbance of the MB solution at 664 nm was best matched with the spectral responsivity of the *R* value rather than those of the *G* or *B* values, as shown in Figure 1c,d. It can also be understood that the orange is close to red, as represented with the *R* value. Thus, the MB reduction rate (MBR*_R_*%) using the *R* value of the RGB photodetector can be calculated using (2):

(2) $${\mathrm{MBR}}_{R}=\frac{D_{0}-D_{t}}{D_{0}-D_{h}}\;\left(\%\right),$$

where *D*_0_ and *D_t_* are the initial *R* value and the *R* value at time *t*, respectively, and *D_h_* is the *R* value of the DI water, which was almost 11.5 in this system.

The MBR*_A_* values were closely correlated with the MBR*_R_* values, as shown in Figure 1e. This suggested that the colorimetric response of the MB reduction to UVC radiation could be presented numerically using the commercially available, low-cost RGB photodetector with the MBR*_R_* parameter. Thus, unless otherwise stated, the colorimetric response of the MB solution was evaluated in the form of MBR*_R_* in this work. 

### 2.4. Kinetic Model Analysis for TEOA Effects on the MB Reduction with Ga_2_O_3_

The TEOA effects on the MB reduction upon UVC exposure were evaluated using pseudo-first-order kinetics (according to the Langmuir–Hinshelwood mode) with (3) [15]:

(3) $$\ln\left(\frac{C_{t}}{C_{o}}\right)=-kt,$$

where *k* is a photocatalytic reaction rate constant (in min^−1^) and $C_{0}\;\left(=D_{0}-D_{h}\right)$ and $C_{t}\left(=D_{t}-D_{h}\right)$ are the initial concentrations of the MB solution and the concentration at time *t* of the UVC irradiation, respectively. The photocatalytic kinetic rate constant (*k*) was obtained by fitting the kinetic traces with linear fittings in the plot of $-\ln\left(\frac{C_{t}}{C_{o}}\right)$ versus time (*t*).

It is worth mentioning that the dynamic equilibrium of the adsorption and desorption kinetics plays a critical role in determining the photocatalytic efficiency [19]. In this work, a change in the absorption spectra of the MB solution with Ga_2_O_3_ photocatalysts was not noticeable within 1 h, while it was significant under UVC exposure. This indicated that the effect of the dynamic equilibrium had less of an impact on MB reduction with Ga_2_O_3_ under UVC with a hole scavenger.

## 3. Results and Discussion

**Figure 2:**
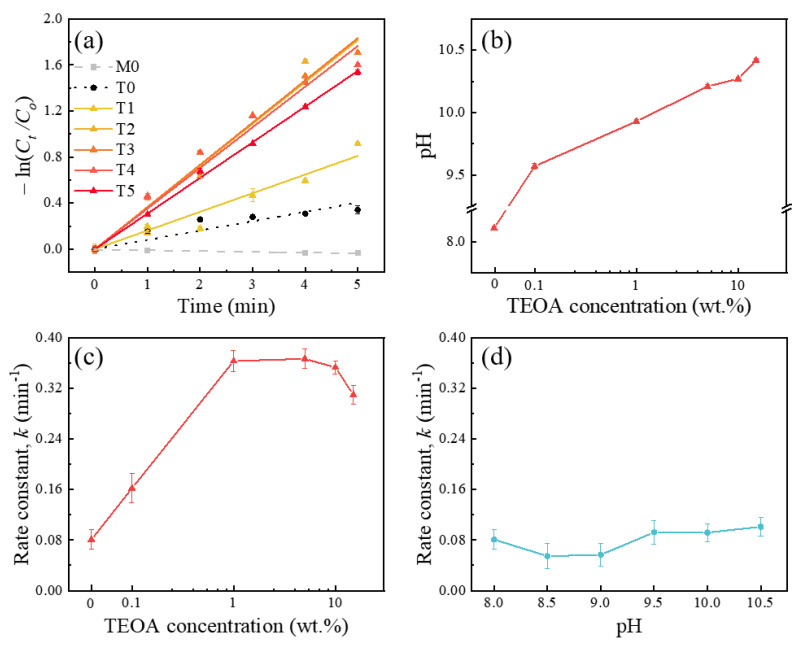
(**a**) Photocatalytic degradation kinetics with a linear fitting for MB reduction at different TEOA concentrations (T0–T5), including without TEOA and Ga_2_O_3_ (M0). (**b**) pH value of the MB solution as a function of the TEOA concentration. The rate constant *k* (min^−1^) of MB reduction as a function of (**c**) the TEOA concentration (T0–T5) and (**d**) the pH changes (8.0–10.5) induced by the addition of ammonia solution.

Figure 2a shows the pseudo-first-order kinetics of the MB reduction with different TEOA concentrations (0–15 wt.%). For comparison, the M0 and T0 were also included as a control set of experiments because the MB itself could be slightly reduced upon UVC exposure [16]. Table 1 includes the specific sample notation for the different TEOA concentrations, along with the linear regression coefficient (coefficient of determination, *R*^2^) obtained from Figure 2a. As the TEOA concentration increased, the pH value of the MB solution increased, as shown in Figure 2b. It was reported that the photocatalytic reaction increased with increasing pH values because OH^−^ ions can form OH· radicals that eventually promote photocatalytic reactions [20,21]. Thus, to distinguish between the scavenger and pH effects on the photocatalytic reactions when adding TEOA in the MB solution, the pH effect on the MB degradation was investigated at different pH conditions (8.0–10.5) by adding NH_4_OH. Figure 2c,d exhibits the rate constant (*k*, min^−1^) for the photocatalytic reduction of MB solution as a function of the TEOA concentration (T0–T5) and the pH value (8.0–10.5) without the addition of TEOA. It shows that the rate constant (*k*, min^−1^) values were significantly affected by the amount of TEOA in the MB solution while it was hardly affected by the pH value in the range of 8.0–10.5. These indicated that the changes in the rate constant (or the MB reduction) were dominated by the TEOA effect rather than the pH changes. It is worth noting that in the previous report, the reaction rate constant (*k*) increased with increasing pH values in the pH range below 8 and reached a plateau in the pH range of 8–10 [21]. A detailed observation of Figure 2c revealed that the rate constant continued to increase as the TEOA concentration increased and then the values decreased as the TEOA concentration was further increased.

**Figure 3:**
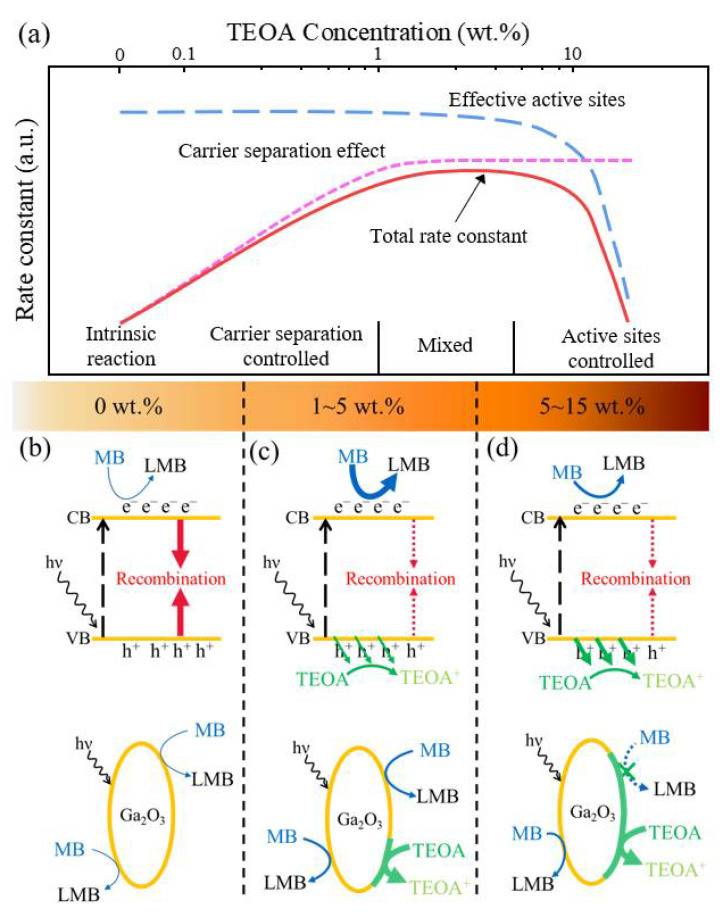
(**a**) Schematic drawing of the rate constants’ behavior, which considers two different effects: carrier separation and the number of effective active sites. The photocatalytic reduction mechanism of the MB solution with the Ga_2_O_3_ photocatalyst (**b**) without TEOA, (**c**) with a low TEOA concentration (1–5 wt.%), and (**d**) with a high TEOA concentration (over 5 wt.%); TEOA^+^ is an oxidized form of TEOA after scavenging a hole. CB: conduction band, VB: valence band, LMB: leuco-MB.

The photocatalytic reduction mechanism of the MB solution with the Ga_2_O_3_ photocatalyst at different TEOA concentrations is further represented in Figure 3. It was presumed that the reduction rate depending on the TEOA concentrations was governed by two different effects: one was the carrier separation by ions and the other was the effective active site. Upon UVC exposure, the Ga_2_O_3_ photocatalyst was excited to generate EHPs, as shown in Figure 3b. The photogenerated EHPs within the Ga_2_O_3_ photocatalysts can be rapidly relaxed either through radiative or nonradiative recombination, which decreases the overall photocatalytic efficiency. The carrier separation is known to effectively suppress the charge recombination, thereby enhancing the overall photocatalytic efficiency.

By adding a small amount of TEOA of less than 1 wt.%, the overall photocatalytic efficiency was enhanced due to the charge separation effect, as shown in Figure 3c. However, at an excessive TEOA level of over 5 wt.% in the MB solution, the TEOA reaction became more dominant on the Ga_2_O_3_ surface rather than the MB reduction, which blocked the catalytically active site on the surface of the Ga_2_O_3_, as shown in Figure 3d. This was because of the chelating nature of the three alcohol groups (–OH) of the TEOA molecules that facilitated the chemical coordination on the Ga^3+^ site on the surface of Ga_2_O_3_, thereby prohibiting chemical reactions between the MB molecules and catalytic sites.

**Figure 4:**
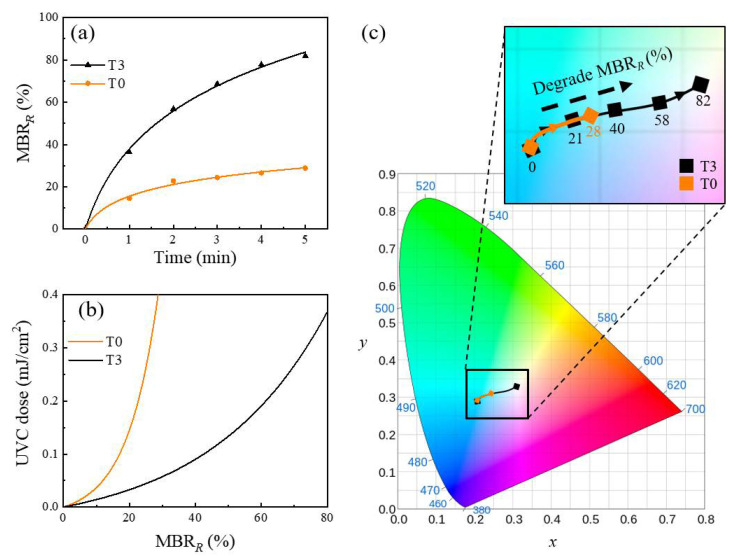
(**a**) MBR*_R_* of T0 and T3 as a function of the UVC irradiation time (0–5 min). (**b**) Accumulated UVC dose (mJ/cm^2^) as a function of the simulated MBR_*R*_ values depending on T0 and T3; the intensity of the UVC radiation was 1.38 mW/cm^2^. (**c**) MBR*_R_* changes of T0 and T3 in the International Commission on Illumination (CIE) chromaticity diagram after 5 min of UVC exposure.

Figure 4a shows the MBR*_R_* of T0 and T3 as a function of the UVC irradiation time. The MBR*_R_* values increased as the MB molecules were chemically reduced. This shows that adding 5 wt.% TEOA (T3) in the MB solution expedited the MB reduction compared with T0, which revealed that TEOA served as a hole scavenger for the Ga_2_O_3_ photocatalysts in the MB solution. Based on the extracted MBR*_R_* values in Figure 4a, an accumulated UVC dose (mJ/cm^2^) could be estimated for T0 and T3, which is shown in Figure 4b. The MBR*_R_* value of T3 changed more significantly than that of T0 at a given dose of the UVC radiation (mJ/cm^2^), indicating that the UVC colorimetric sensor with T3 showed an improved responsivity compared with that when using T0. The colorimetric responses of T0 and T3 for the UVC exposure were visualized and compared on the standard CIE chromaticity diagram in Figure 4c; upon 5-min UVC exposure, the MBR*_R_* of the T3 changed from 0 to 82%, while that of T0 changed from 0 to 28%. 

## 4. Conclusions

A highly responsive UVC colorimetric sensor was demonstrated by adding less than 5 wt.% TEOA to an MB solution with a Ga_2_O_3_ photocatalyst. The small amount of TEOA in the MB solution served as a hole scavenger and thereby enhanced the charge separation, leading to fast MB color changes. However, excessive TEOA over 5 wt.% in the MB solution started to block the active catalytical site on the surface of the Ga_2_O_3_, which prohibited chemical reactions between the MB molecules and catalytic sites. Unlike conventional color recognition using ex situ UV-Vis spectroscopic measurements, the MB color changes were monitored using in situ *R* values from RGB photodetectors. The MB reduction obtained from the in situ RGB photodetector sensor was linearly correlated with conventional ex situ UV-Vis spectroscopic measurements. This work demonstrated that the colorimetric UVC sensor could monitor the detrimental UVC radiation with high responsivity and low cost. Furthermore, the data was digitalized in real time, which is beneficial for connecting to Internet-of-things (IoT) technology.

## Figures and Tables

**Table 1:** Experimental conditions with different TEOA concentrations.

Index	M0	T0	T1	T2	T3	T4	T5
Ga_2_O_3_ (mg)	0	9	9	9	9	9	9
TEOA (wt.%)	0	0	0.1	1	5	10	15
*R*^2^ *	0.97	0.95	0.98	0.99	0.99	0.99	1.00

* *R*^2^: the linear regression coefficient (coefficient of determination).
